# Fruit growth and development in apple: a molecular, genomics and epigenetics perspective

**DOI:** 10.3389/fpls.2023.1122397

**Published:** 2023-04-12

**Authors:** Khalil R. Jahed, Peter M. Hirst

**Affiliations:** Department of Horticulture and Landscape Architecture, Purdue University, West Lafayette, IN, United States

**Keywords:** MdCNR, quantitative trait loci, fruit growth regulation, pollination, double fertilization, epigenetics, cyclin dependent kinase, hormone

## Abstract

Fruit growth and development are physiological processes controlled by several internal and external factors. This complex regulatory mechanism comprises a series of events occurring in a chronological order over a growing season. Understanding the underlying mechanism of fruit development events, however, requires consideration of the events occurring prior to fruit development such as flowering, pollination, fertilization, and fruit set. Such events are interrelated and occur in a sequential order. Recent advances in high-throughput sequencing technology in conjunction with improved statistical and computational methods have empowered science to identify some of the major molecular components and mechanisms involved in the regulation of fruit growth and have supplied encouraging successes in associating genotypic differentiation with phenotypic observations. As a result, multiple approaches have been developed to dissect such complex regulatory machinery and understand the genetic basis controlling these processes. These methods include transcriptomic analysis, quantitative trait loci (QTLs) mapping, whole-genome approach, and epigenetics analyses. This review offers a comprehensive overview of the molecular, genomic and epigenetics perspective of apple fruit growth and development that defines the final fruit size and provides a detailed analysis of the mechanisms by which fruit growth and development are controlled. Though the main emphasis of this article is on the molecular, genomic and epigenetics aspects of fruit growth and development, we will also deliver a brief overview on events occurring prior to fruit growth.

## 1 Introduction

Fruit growth and development are biological processes that form a complex regulatory machinery, which are controlled by a multitude of sequential events. The successful accomplishment of such process determines final fruit size, one of the most important quality parameters defining the marketability of fruit. These developmental processes have been studied extensively at both physiological and molecular levels. Several regulatory factors and genes underlying these processes have been identified and their regulatory mechanisms have been investigated. Additionally, the rapid advances in high-throughput sequencing technology combined with improved statistical and computational methods have allowed scientists to efficiently sequence the whole genome of apple and the resultant information can help dissect complex traits such as fruit size and identify genomic regions regulating such traits. Enormous amounts of data are available, especially for traits related to fruit quality and yield improvement (Eccher et al., 2014). Furthermore, the availability of whole-genome sequencing over the past decade has facilitated increased understanding of biological functioning of many traits includingthose related to yield, fruit growth and development, and epigenetic aspects of fruit size, including domestication, leading to immediate practical implications for improving breeding programs (Eccher et al., 2014; Peace et al., 2019). Such advances combined with the economic importance of apple as a crop have made apple (Malus x domestica) an emerging model for fruit development studies (Eccher et al., 2014). The aim of this article is to provide a chronological narrative of the physiological processes that mediate fruit growth, and to summarize the current knowledge of molecular and genomic regulation of fruit growth and resultant effects on fruit size. The review also provides a comprehensive narrative of approaches by which quantitative traits such as fruit size are dissected. Additionally, obstacles associated with dissecting such a complex, multifactorial inherited, and polygenic trait as well as specific recommendations are discussed. Finally, future research directions to enrich our knowledge are suggested at the end of this article.

## 2 Flower induction, initiation, and differentiation

Apple produces mixed buds composed of both vegetative and reproductive components. The process of producing an apple fruit begins with the transition of a bud from a vegetative to floral state. Such buds are induced during the previous growing season as early as three to six weeks after bloom (Buban and Faust, 1982), which is the result of a series of internal developmental events occurring in a chronological order (Wilkie et al., 2008). The transition from a vegetative to floral state involves increased complexity of the bud apex (Hirst and Ferree, 1995) accompanied by major changes in the pattern of histogenesis, morphogenesis and cell differentiation at the shoot apical meristem (Buban and Faust, 1982; Jackson, 2003). These changes are triggered by increased synthesis of nucleic acids and histone modification within the vegetative apex (Jackson, 2003). Cellular differentiation at the apex continues through the growing season. However, it temporarily ceases during winter dormancy. During winter, floral buds undergo endodormancy and the flowers are completed after overcoming endo- and subsequently eco-dormancy (Sung et al., 2000).

Progress has been made in the understanding of genetic and molecular regulation of flower bud development. Several floral regulatory integrator genes have been identified. These genes are *FLOWERING LOCUS T* (*FT*), *SUPPRESSOR OF OVEREXPRESSION OF CONSTANS1* (*SOC1*)/*AGAMOUS LIKE20*, *FLOWERING LOCUS C* (*FLC*), and *TERMINAL FLOWER1* (*TFL1*). These integrators transmit signals to the floral meristem-identity genes, *APETALA1* (*AP1*) and *LEAFY* (*LFY*), at the apical meristems (Araki, 2001; Jack, 2004; Michaels, 2009). Of these, *FT*, *SOC1*, *AP1* and *LFY* promote flowering, and their overexpression causes early flowering, whereas *FLC* and *TFL1* suppress flowering, and their overexpression delays flowering (Li et al., 2010). *FT* is regulated *via* autonomous, photoperiod (Kobayashi et al., 1999), and vernalization transduction pathways (Mimida et al., 2011). Its photoperiodic induction is triggered by the zinc finger protein, *CO*. This gene is repressed by *FLC*, a vernalization integrator, and *TFL1* flowering repressor (Michaels and Amasino, 1999; Kotoda et al., 2000; Takada and Goto, 2003). *LFY* and *AP1*, the floral meristem-identity genes, mediate the transition of flower buds from vegetative to reproductive phase and promote flowering in conjunction with *FT* gene (Schultz and Haughn, 1991; Weigel et al., 1992). *FLC* is an important repressor of flowering and is regulated by both autonomous and vernalization pathways. This gene inhibits flowering by suppressing the expression of the floral pathway integrators *CO*, *LFY*, *SOC1*, and *FT* (Helliwell et al., 2006), through a rheostat-like mechanism alongside another inhibitor, *TFL1* (Kardailsky et al., 1999; Kobayashi et al., 1999; Michaels and Amasino, 1999).

In apple, an increased expression of two *FT*-like orthologs genes, *MdFT1* and *MdFT2*, promoted early flowering (Li et al., 2010), each exhibiting distinct expression pattern. The *MdFT1* gene expresses in apical meristematic tissue of fruit-bearing shoots during the transition period from vegetative to reproductive phase, whereas *MdFT2* expresses in reproductive organs, including flower buds. The differential expression of these genes is proposed to imply distinct function, with *MdFT1* promoting flowering and *MdFT2* integrating reproductive organs development (Mimida et al., 2011). Such evidence of *MdFT1* promoting flowering was further supported by its overexpression in a transgenic apple line that resulted in an extremely early-flowering phenotype (Kotoda et al., 2010) and downregulation of *FT1* by defoliation treatments which decrease flowering (Elsysy and Hirst, 2019). Molecular characterization of *MdFT1* and *MdFT2* transcripts showed that these genes are involved in the regulation of cellular proliferation and formation of new tissue that further affect organ development by interacting with two members of the apple TCP (TEOSINTE BRANCHED1, CYCLOIDEA and PROLIFERATING CELL FACTORs)-like family and one member of the apple VOZ (Vascular plant One Zinc finger protein)-like family proteins (Mimida et al., 2011).

Similarly, two orthologs of the *Arabidopsis FLC* gene were identified in apple, *MdFLC1* and *MdFLC3* (Kagaya et al., 2020). The reduced expression levels of three subsets of *MdFLC1, MdFLC1a, MdFLC1b*, and *MdFLC1c* during floral bud induction at a seasonal expression pattern, suggested that *MdFLC1* induces flowering, whereas *MdFLC3* is suggested to function as floral repressor (Kagaya et al., 2020). These data indicate that flower induction in apple is controlled by a complex network that is tightly regulated by both an internal signaling cascade and environmental stimuli.

## 3 Pollination and double fertilization

Pollination is the process of transferring pollen grains from anther, the male organ of the flower, to the stigma of the pistil, the female organ of the flower (Taiz et al., 2015). A mature pollen grain is composed of a large vegetative/tube cell and two sperm cells (McCormick, 1993; Raghavan, 2006). The delivery of sperm cells from the stigmatic surface to the ovary is mediated by a pollen tube, which occurs in six consecutive phases.

(1) Pollen grain adhesion, hydration, and germination on the stigmatic surface of the pistil. In apple, the stigma has a wet surface where the papilla cells release extracellular secretions, typically composed of a mixture of proteins, lipids and polysaccharides that provide moist environments for pollen germination (Heslop-Harrison, 1976; Sedgley, 1990).

**(**2) The germinated pollen grains on the stigmatic surface generate pollen tubes penetrating the stigmatic surface and grow toward the transmitting tissue within the intercellular space.

(3) The pollen tube continues to grow through the nutrient-rich interstitial material of the transmitting tissues (Jackson, 2003). Transmitting tissues provide nutrients and mechanical support to the growing pollen tube. These tissues then degenerate, providing extra space for pollen tube penetration within the style.

(4) The growing pollen tube exits transmitting tissue and grows toward the ovule, which is guided by two ovular guidance mechanisms, funicular- and micropylar-guidance (Dresselhaus and Franklin-Tong, 2013). These mechanisms are actively involved in the next two phases.

(5) Once the pollen tube reaches the ovule, it grows at the surface of the septum and the funiculus toward the micropyle, the entrance of the ovule.

(6) Finally, the pollen tube passes the micropyle, penetrates the egg apparatus, and enters one of the synergid cells, which is guided by micropylar guidance mechanism (Drews and Yadegari, 2002). The receptive synergid cell degenerates and undergoes cell death, and the tip of pollen tube ruptures to release the two sperm cells (Rotman et al., 2003; Sandaklie-Nikolova et al., 2007; Drews and Koltunow, 2011; Hamamura et al., 2012; Dresselhaus and Snell, 2014).

Several peptide/receptor-like kinase-mediated signaling cascades (PRKs) have been identified that control these phases. Functional analysis of these cascades showed that they promote pollen-stigma interaction, pollen adhesion and hydration, and pollen-tube growth down the style (Zhong and Qu, 2019). Pollen tube communication with synergid cells, a critical constitute of pollen tube reception, is controlled by a group of CRPs including rapid alkalization factors (RALFs), and their corresponding receptors, such as RLK1-LIKE and FERONIA (FER)/SIRENE Escobar-Restrepo et al., 2007; Ge et al., 2019; Liang and Zhou, 2018. FER is a synergid-specific signaling cascade expressed at the filiform apparatus, the first structure that communicates with a pollen tube. This receptor is likely involved in the crosstalk between the arriving pollen tube and the receptive synergid cells, particularly during the pollen tube growth arrest (Zhong and Qu, 2019).

After the sperm cells are discharged from the pollen tube, double fertilization occurs, in which one sperm cell fertilizes the egg cell to form a diploid embryonic zygote, and the second sperm cell fuses with the central cell nuclei to form a triploid endosperm cell (Berger et al., 2008; Drews and Koltunow, 2011; Dresselhaus and Snell, 2014). The main function of the endosperm cell is to provide nutrients and supply resources for the embryonic zygote during its initial heterotrophic phase. Successful fertilization of an egg cell within the ovary depends on several factors such as temperature, genetic compatibility (Jackson, 2003), and the interaction of pollen grain with stigmatic surface, as well as with transmitting that is mainly controlled by compatibility reactions (Pratt, 1988). Despite the extensive communication between the male and female gametes, few factors have been identified to be involved in their interaction. A sperm-specific candidate gene, GAMETE EXPRESSED2 (GEX2), which contains an extracellular immunoglobulin-like domain, is involved in gamete recognition and attachment (Mori et al., 2014). Two other domains (DUF679) DMP8 and DMP9/DAU2 have been identified to facilitate gamete fusion, particularly the sperm-egg fusion (Takahashi et al., 2018; Cyprys et al., 2019). Additionally, a group of five CRP peptides, EGG CELL1s (EC1s) and an egg-localized fusion protein, EC1-GFP, are extensively involved in gamete activation (Zhong and Qu, 2019), and sperm adhesion and sperm-cell separation (Cyprys et al., 2019).

**Figure 1:**
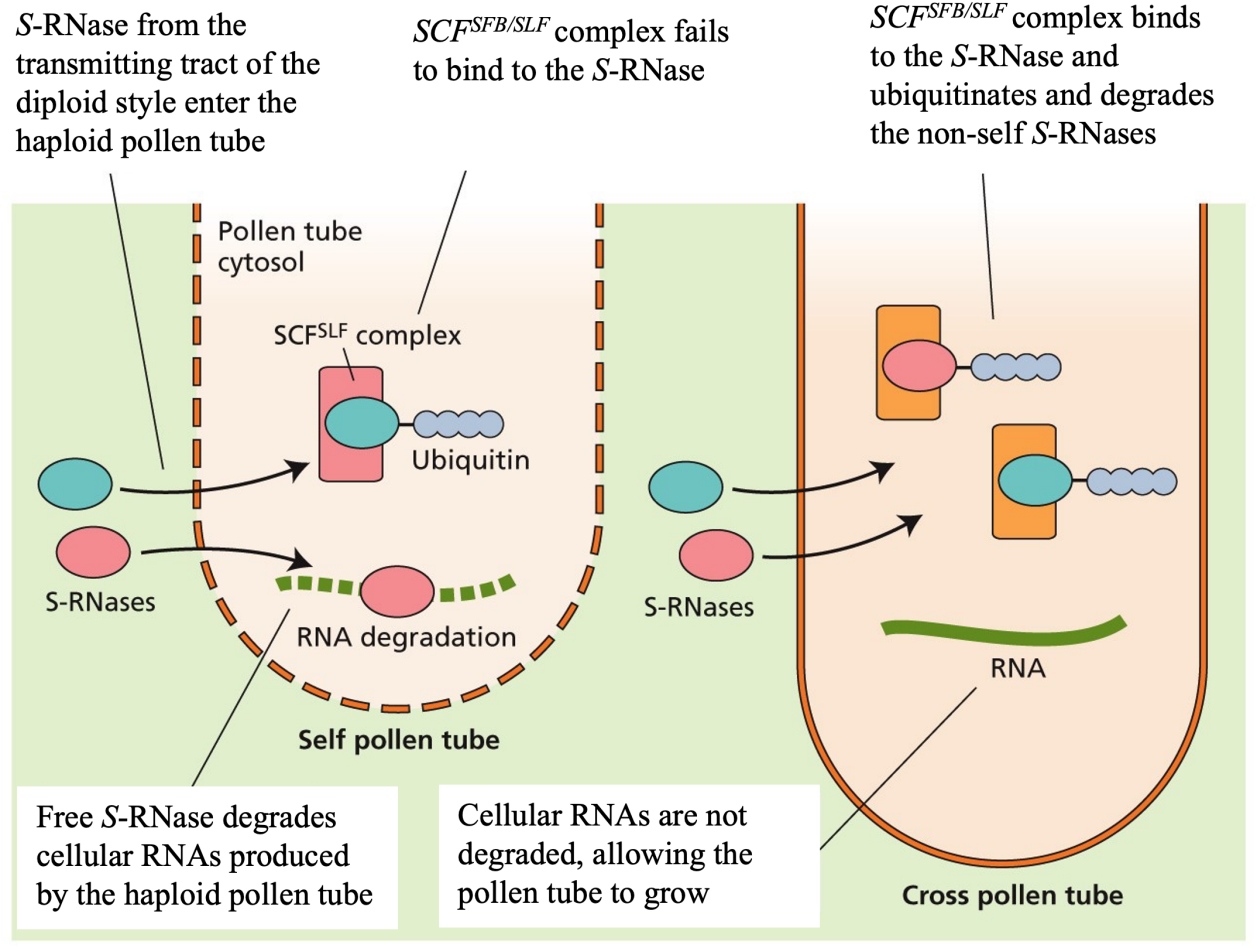
Molecular mechanism of gametophytic self-incompatibility (GSI). The pistil Sdeterminant is specified by cytotoxic S-glycoproteins or *S-RNase* gene expressed in the transmitting tissue of the style. S-RNase from the diploid style enters the haploid pollen tube. In cross-pollinated flowers (right in the Figure), the *SCF^SFB/SLF^* complex binds to the S-RNase, which ubiquitinates and degrades the non-self S-RNases. Such degradation of the S-RNase prevents cellular RNAs degradation caused by S-RNase, allowing the pollen tube to grow. In self-pollinated flowers (left in Figure), the *SCF^SFB/SLF^* complex fails to bind to the S-RNase, resulting in cellular RNAs degradation by the free S-RNase, leading to block pollen tube growth. Figure is reproduced with permission of the Licensor through PLSclear.

Generally, apple reproduces *via* cross-pollination/outcrossing norm – where the male and female parents are separate sporophytic individuals. Apple is self-incompatible, where the pollen grain is incapable of fertilizing its own egg cell. Multiple incompatibility mechanisms have been identified in flowering plants. Of these, gametophytic self-incompatibility (**GSI**) appears to be the predominant mechanism in apples. The incompatibility recognition in the **GSI** system occurs in the transmitting tissue and is mediated by a single multiallelic locus (locus ***S***) containing two tightly linked genes, one encoding pollen-expressed male *S-*determinant and the other regulates pistil-expressed female *S-*determinant (Jackson, 2003; Ramírez and Davenport, 2013; Seymour et al., 2013; Wu et al., 2013). The pollen *S-*determinant is specified by a highly polymorphic gene, *SFB/SLF*, expressed in pollen. *SFB/SLF* is a F-box protein, which is involved in binding target proteins into the *SCF* complex using E3 (ubiquitin-ligase) through the polyubiquitin-26S proteasome-dependent pathway. The pistil *S-*determinant is specified by cytotoxic *S*-glycoproteins or *S*-RNases gene expressed in the transmitting tissue of the style where the incompatibility recognition occurs (Dresselhaus and Franklin-Tong, 2013). In a cross-pollinated flower, *S*-RNase taken up from the transmitting tissue of the diploid style enters the haploid pollen tube that grows down the style and the *SCF^SFB/SLF^* complex binds to the *S*-RNase, which ubiquitinates and degrades the non-self *S*-RNases. Such degradation prevents self-RNase cytotoxicity, allowing the pollen tube to continue growing (**Figure 1**). In a self-pollinated flower, the *SCF^SFB/SLF^* complex fails to bind to the *S*-RNase of the transmitting tissue. As a result, the free *S*-RNase degrades cellular RNAs produced by the haploid pollen tube, leading to inhibition of the pollen tube growth (Dresselhaus and Franklin-Tong, 2013) (**Figure 1**).

**Figure 2:**
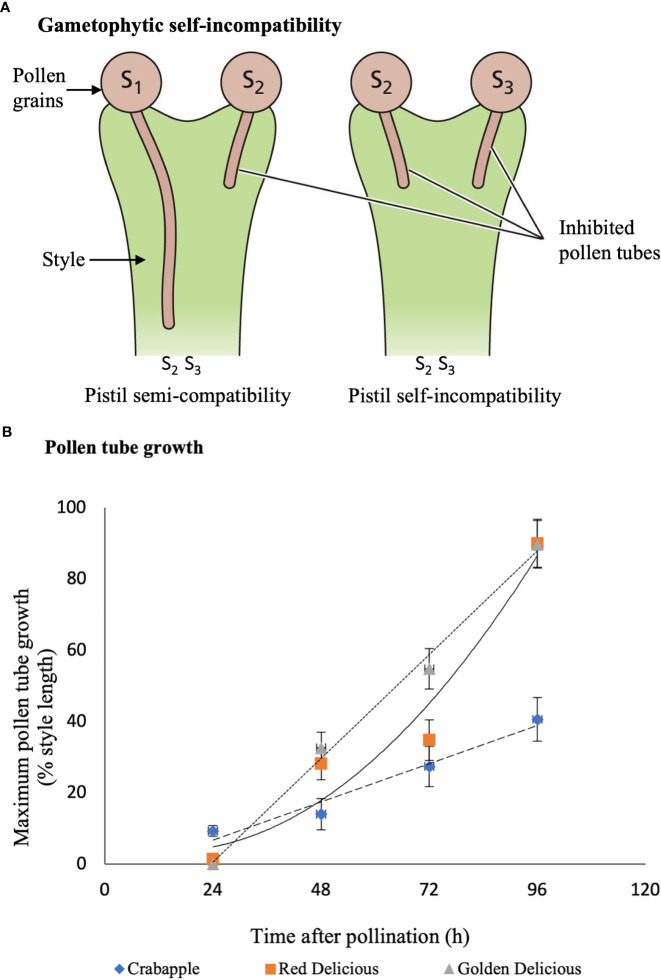
Semi-incompatibility in apple. **(A)** represents the gametophytic semi-incompatibility mechanism compared with self-incompatibility. During self-incompatibility, both pollen grain *S-haplotypes* are identical to those of the pistil, whereas in semiincompatible flowers, only one pollen grain S-haplotype matches one of the pistil Shaplotype. **(B)** represents the pollen tube growth of ‘*Malus floribunda*’ crabapple, ‘Red Delicious’, and ‘Golden Delicious’ pollen crossed with ‘Honeycrisp’ apple. Crabapple has the slowest growth rate. **(A)** is reproduced with permission of the Licensor through PLSclear, while **(B)** is data from our previous work (Jahed and Hirst 2017).

Though the majority of apple species display self-incompatibility, semi-compatibility has become more prevalent as more interrelated cultivars are grown (Matsumoto, 2014; Jahed and Hirst, 2017). Pollen grains of these species are capable of fertilizing the ovule during double fertilization; however, a much lower percentage of fruit might have set (Jahed and Hirst, 2017). We conducted a series of pollination studies, that were previously published (Jahed and Hirst, 2017), where six apple cultivars were hybridized in 2013 and repeated in 2014. The results showed that less than seven percent of hand-pollinated flowers produce fruits when ‘Honeycrisp’ cultivar was pollinated by ‘*Malus floribunda*’ crabapple. The pollen tubes had the lowest germination and slowest growth in ‘Honeycrisp’ stigmas and styles, and comparatively, fewer pollen tubes reached the base of the style (**Figure 2B**). This may have been a result of semi-compatibility of ‘*Malus floribunda*’ crabapples with ‘Honeycrisp’, which, resulted in a lower fruit set (Jahed and Hirst, 2017), and fewer seeds per fruit (Jahed and Hirst, 2018). In such **GSI** systems, one of the pollen grain *S-*haplotypes matches either of the pistil *S-*haplotype and rejection occurs, where the second pollen *S-*haplotype differs from both of the pistil *S-*haplotypes, thus, the pollen tube continues to grow (Matsumoto, 2014) (**Figure 2A**).

## 4 Fruit set

Soon after pollination and ovule fertilization, the ovary and surrounding receptacle tissues begin to grow. The ovary tissue development continues through rapid cell division, cell enlargement, and intracellular space (voids) production leading to fruit set (Lakso and Goffinet, 2017). The endosperm nucleus, derived from the fusion of a sperm cell and the two polar nuclei, undergoes a series of several mitotic divisions and about 4-6 weeks after fertilization, it becomes cellular (Ferree and Warrington, 2003). It has been thought that fruit set is tightly controlled both by the interaction between phytohormones such as auxin and gibberellin, and by the expression of transcripts that trigger cell production (Fenn and Giovannoni, 2021). Of these, many of the positive cell cycle regulators such as the A- and B-type CYCLINS, and several CYCLIN DEPENDENT KINASES-B (CDKBs) showed an increased transcriptional accumulation during fruit set. However, negative regulators such as the CKD inhibitor, KRP4, showed reduced transcript accumulation during this period (Malladi and Johnson, 2011). Further, floral homeotic genes such as MADS-box genes, are involved in the growth of the ovary and surrounding receptacle tissues. These genes trigger floral organs development through tightly controlled genetic regulation and are involved in the regulation of fruit set.

In general, apple fruit development can be divided into two distinct stages: **(1)** early developmental stage, and **(2)** late developmental stage (Eccher et al., 2014). The former is divided into three sub-developmental phases: the first sub-phase involves floral initiation, carpel formation, and pre-anthesis cell division that leads to ovary growth and ends with anthesis. The second sub-phase includes pollination, double fertilization, fruit set, and the restart of post-anthesis cell division. A third sub-phase encompasses cell expansion, which leads to an increase in cell size and subsequently fruit size (Okello et al., 2015). The late developmental stage includes fruit maturity and ripening (Eccher et al., 2014). Each stage is controlled by a complex network of both internal and external factors. Among the former, the role of phytohormones on the transition from flower to fruit is pivotal and well established, at least in model plants such as *Arabidopsis* and tomato (Molesini et al., 2020). Several phytohormones including auxin, gibberellins (GAs), cytokinins (CKs), abscisic acid (ABA), ethylene, and brassinosteroids (BRs) play important roles in different stages of fruit development – from fruit set to fruit ripening. Of these, auxin and GAs promote the initiation of fruit set through a crosstalk (Serrani et al., 2008; de Jong et al., 2009; Fenn and Giovannoni, 2021). Such auxin-GA communication is facilitated by the action of Aux/IAA, and auxin response factor (ARF) proteins like ARF106 (Devoghalaere et al., 2012). ARF106 gene co-segregates with a strong QTL controlling fruit size and regulates fruit weight through the auxin signaling transduction pathway.

At the molecular level, auxin inactivates the repressor complex that blocks ovary growth before pollination and fertilization. Immediately after pollination and fertilization, the auxin content within the ovary increases leading to the activation of the auxin signaling pathway, which initiates fruit set (Salehin et al., 2015). Another contribution of auxin to the regulation of the fruit set is inducing GA accumulation in the ovary, by upregulating GA biosynthesis genes (Serrani et al., 2008; McAtee et al., 2013; Kumar et al., 2014). This activates the GA-signaling pathway leading to the degradation of DELLA protein, an organ growth-repressor. Apple displays inconsistent response to exogenous application of phytohormones. For instance, exogenous applications of GA promotes parthenocarpic fruit in apple (Malladi, 2020), whereas such fruits are induced by applying exogenous applications of auxin and GA in other crops such as tomato (Serrani et al., 2007; An et al., 2020). Together, these data illustrate that phytohormone, particularly auxin and GA, and auxin/GA-responsive genes are actively involved in the initiation of fruit set.

## 5 Fruit growth

### 5.1 The cellular components of fruit growth

In apple, fruit growth is mediated primarily by the process of cell division, cell expansion, and the development of intercellular spaces (the void space). In other fleshy fruit species, for instance tomato, endoreduplication (an increase in the nuclear genome without mitosis) is another constituent of fruit growth. The relative contribution of these interrelated processes to fruit growth depends on the plant species under consideration. Cell number is commonly accepted to be positively correlated with fruit growth and final fruit size in all fleshy-fruit species (Harada et al., 2005; Johnson et al., 2011; Okello et al., 2015); however, cell size and endoreduplication do not always display a linear correlation. Cell number in this review refers to the total cells produced during the active cell division period at both pre- and post-anthesis stages. Cell size, on the other hand, refers to the maximum volume a cell obtains after the period of cell expansion. In apple, increased cell size within the cortex tissues was responsible for fruit size differences between two apple cultivars, ‘Gala’ and an endoreduplicating mutant, ‘Grand Gala’ (Malladi and Hirst, 2010). The authors noted that ‘Grand Gala’ displayed larger cortex area, larger cell size, and higher ploidy level, whereas cell number remained consistent between the two cultivars. This was the first, and perhaps the only finding of endoreduplication in apple. Meanwhile, these findings appear to be based on the proportion of large cells in the two cultivars studied, rather than the average cell size. Thus, a detailed and comprehensive study to compare the average cell size is needed. In other plant species (i.e., tomato) endoreduplication is reported to be the second-most contributing factor, after cell number, influencing fruit growth and final fruit size, whereas cell size tends to have little influence on these events (Okello et al., 2015).

In apple fruit, cell division begins in the previous growing season as early as the floral buds are induced and initiated (Buban and Faust, 1982), and continues until approximately 35 days after full bloom (DAFB) (Janssen et al., 2008). The early cell division phase appears to be tightly regulated by genes involved in cell-cycle regulation pathways (Janssen et al., 2008). During this rapid cell division phase, nucleus and endosperm grow rapidly while the embryo has much slower developmental speed. At this phase, the control of cell cycle and energy supply are critical because their deficiency might have a negative effect on fruit quality or might trigger fruitlet abscission (Eccher et al., 2014). Additionally, pre-anthesis cell division, size of the ovary at anthesis, post-anthesis cell division (Okello et al., 2015), and seed number per fruit (Jahed and Hirst, 2018) appear to influence fruit size. In addition to the direct correlation of seed and final fruit size, seeds are postulated to be the source for phytohormones such as cytokinins and auxins, which stimulate cell division and cell expansion in developing ovaries. Seeds also regulate the flow of assimilates and nutrients within the fruit, as well as affect fruit shape, which depends on the distribution of seeds within the locules (Eccher et al., 2014).

Cell division is followed by cell expansion during which cells obtain their maximum volume and the fruit accumulates metabolites and energy in the form of starch. The duration of the cell expansion phase is much longer than that of cell division which continues until shortly before the fruit attains its full size (Janssen et al., 2008). Cell expansion displays a high initial rate followed by a rapid decline in about 45 DAFB in small-fruited crabapple (Harada et al., 2005), and 60 DAFB in large-fruited domesticated cultivars, but continues at a reduced rate until fruit reaches the ripening stage (Janssen et al., 2008). This reduction is typically followed by a slight increase in the expansion rate during the late fruit development stages (Dash and Malladi, 2012; Dash et al., 2013). Subsequently, cell expansion (usually after seeds mature and fruits obtain maximum volume) is followed by a series of biochemical changes during which the stored energy (i.e., starch) is converted to more available compounds such as glucose. During this stage, the endosperm becomes cellularized, the embryo achieves its maximum developmental speed to reach maturity, the volatile secondary metabolites are produced that are assumed to function as attractants for animals and insects, and the fruit enlarges exclusively due to cell expansion (Eccher et al., 2014).

Another major contributor to apple fruit growth is the development of void spaces (intercellular spaces); however, its regulation remains poorly understood in spite of its important contribution to fruit growth. Traditionally, histological methods have been used to determine the contribution of voids to fruit growth. Of these, one method is based on the ratio of resistance of fruit tissue to evacuate its contained air to the proportion of intracellular volume (Goffinet and Robinson, 1995). In such a method, the measurements are based on the volume of water displaced as a vacuum is applied above submerged pieces of tissue. A similar method has been developed that estimates voids by weighting fruit submerged in water or various concentrations of sucrose, then vacuum is applied to infiltrate the tissue and the fruit is reweighed (Reeve, 1953). However, these traditional methods are limited to the capacity of air removal from tissue (in the case of the former method), and to reduced infiltration of pores with aqueous (in the later method). Thus, methods with immediate and accurate implications have recently been developed to measure the void space. For instance, a three-dimensional imaging analysis using X-ray micro-Computed Tomography (µCT) technology has been applied to determine voids in apple (Mendoza et al., 2007; Verboven et al., 2008; Herremans et al., 2015), and were extended to other crops such as cucumber (*Cucumis sativus*) (Kuroki et al., 2004), mango (*Mangifera indica*) (Cantre et al., 2014b), and kiwifruit (*Actinidia deliciosa*) (Cantre et al., 2014a). Such advance techniques display improved measurements of voids and their contribution to fruit size.

The relative contribution of voids to fruit growth varies in species under consideration. For example, the total contribution of void space to fruit growth and development was 23% in apple, while this fraction was only 5.1% in pear (Pyrus communis L.) (Verboven et al., 2008). Additionally, void spaces display a tissue-specific distribution such that it is higher in cortex and lower toward the core tissues. Likewise, voids located in core are typically more fragmented, and likely decreases towards the end of maturity (Herremans et al., 2015). Nonetheless, in the cortex, the volume of the voids increases during the fruit developmental processes (Mendoza et al., 2010). Though these advanced technologies have significant impact on the fruit growth measurements, much more needs to be done to integrate these tools with physiological, molecular, and potentially with genomic measurements to develop a better understanding of fruit growth and development (Cieslak et al., 2016).

### 5.2 Molecular regulation of fruit growth

During the early fruit development stage, the cellular components of fruit growth and their metabolism are highly regulated at the transcriptional level (Li et al., 2012; Eccher et al., 2014). These regulations can be classified as acting at the cellular and organ level. *At the cellular level*, a plant cell undergoes several rounds of the mitotic cell cycles, through multiple, sequentially ordered phases leading to the generation of two genetically identical daughter cells (Dewitte and Murray, 2003; Francis, 2007; Harashima and Sekine, 2020). Multiple cell-cycle related transcriptional regulators have been identified that control these processes. Of these, the key components are the regulatory proteins, CYCLINS (CYC), and the special class of serine-threonine protein kinases, which require binding to cyclin protein for activity, CYCLIN DEPENDENT KINASES (CDKs) complexes (Dewitte and Murray, 2003; Inzé and De Veylder, 2006). In plants, unlike single-cellular organisms like yeast (*Saccharomyces cerevisiae*), multiple cyclins have been identified that are involved in different aspects of cell biology and particularly in cell-cycle regulation, specifically in controlling the transition from one cell-cycle phase to another (Joubès et al., 2000; Dewitte and Murray, 2003). For instance, of the five types of cyclins that are classified based on sequence organization (A, B, C, D, and H types) (Renaudin et al., 1996; Vandepoele et al., 2002), A-type cyclins, that appear at the beginning of S phase, control the progression of S-phase, B-type cyclins, which appear during G2-phase, are involved in G2/M and mitotic transitions, and D-type cyclins regulate the transition of G1-S phase (Dewitte and Murray, 2003). Similarly, several types of CDKs have been identified in plants, some of which (e.g., A-type CDK (CDKA), and B-type CDKs (CDKBs)) are abundant, particularly CDKBs, others, such as CDKC, CDKD, and CDKE, form less abundant classes (Joubès et al., 2000).

Additionally, CDK/CYC forms a complex network that is mediated by four regulatory mechanisms: (**1)** binding of CDK to CYC, **(2)** inhibition of CDK through CDK-inhibitory proteins (CKIs), **(3)** the inhibitory phosphorylation of CDK by the conserved residues of its ATP-binding pocket, and **(4)** the activation of CDK through dephosphorylation that is mediated by a conserved residues in its T-loop (Harashima and Sekine, 2020). Many of these cell-cycle machinery components and their regulatory mechanism are conserved in plants, in particular the CDK activity in binding to CYC and their integrative control of the transition among the different phases (Scofield et al., 2014). However, its regulation requires the formation of a multi-level regulatory-complex during molecular development such that forming the heterodimeric protein complexes, as a result of CYCs-CDKs interaction, induces the oscillation of CDK activity during cell-cycle (Harashima and Sekine, 2020). Though the role of cell-cycle machinery components is well-known in cell division, their contribution to organ growth and development remains an arguable subject (Inzé and De Veylder, 2006). Nevertheless, the loss of function of *Arabidopsis* CDKC indicated that the mutation affected the transcription of downstream genes, such as those involved in cell cycle and organ development, which resulted in altered organ size (Harashima and Sekine, 2020). These findings indicate that cell-cycle regulators are key facilitators of organ growth and development.

In apple, multiple genes involved in the cell-cycle machinery have been identified and their expression levels at different developmental stages were investigated (Janssen et al., 2008). The global gene expression analysis indicated that two putative CDKB regulators altered their expression pattern during cell division, suggesting that they are key players of the cell cycle. Additionally, a third member of the cell-cycle machinery, cyclin-dependent kinase subunit1 (CKS1), that changed its expression during fruit development, has been identified, which associates with CDKB protein, and has been involved in activating the regulators that mediate CDK activity (Janssen et al., 2008). Expression analysis of the CKS1 regulator showed that the transcript abundance of this subunit was increased during the cell-division phase (between 7 and 35 DAFB, depending on the cultivar under consideration) (Jiao et al., 2021). In a similar, but detailed study, 71 cell-cycle genes were identified during apple fruit development (Malladi and Johnson, 2011). Of these, 14 genes were found as being positively associated with cell production. Several members of B-type cyclin-dependent kinases and A- and B-type CYCs were included in this group, which suggests the limitation of G2/M phase regulators of the cell cycle during cell proliferation. Meanwhile, five genes including the CDK inhibitors, KRP4 and KRP5, were found to be negatively associated with cell production. Transcriptional analysis of these regulators displayed complementary expression patterns to those of the positive regulators: typically, a reduced accumulation during early developmental stage (normally cell division), and a higher accumulation at the transition phase from cell division to cell expansion (Malladi and Johnson, 2011). Furthermore, the transcriptional abundance of these regulators is greatly influenced by both environmental cues such as temperature and sunlight, and management cues such as crop load and pollination. For instance, the expression levels of the positive regulators showed a reduced accumulation in unpollinated fruit, and in response to high temperature and severe shading (Malladi and Johnson, 2011; Dash et al., 2012; Flaishman et al., 2015), and an increased accumulations in manually thinned fruits (reduced crop load) during the fruit-development phase (Dash et al., 2013). In contrast, the negative regulators, such as KRPs, displayed increased transcript abundance in unpollinated fruit, and under high temperature and severe shading (Malladi and Johnson, 2011; Dash et al., 2012; Flaishman et al., 2015), resulting in reduced cell production.

*At the organ level*, multiple organ-related genes have been identified in higher plants and their regulation of organ size has been investigated. One such gene that is involved in the regulation of organ growth is *AINTEGUMENTA (ANT)* (Elliott et al., 1996). *ANT* is a member of the plant-specific APETALA2/ETHYLENE RESPONSE FACTOR (APT2/ERF) – domain family of transcriptional factor genes – a group of genes that mediate the floral organ identity (Mizukami and Fischer, 2000). *ANT* regulates cell production and displays increased expression levels during early organ growth stages that coincides with cell division; thus, it has been thought to be involved in enhancing organ growth (Mizukami and Fischer, 2000; Dash and Malladi, 2012). *Arabidopsis ant* mutant lines exhibited a reduced cell production rate that resulted in organ size reduction, while overexpression of this gene showed increased organ size, mainly by extending the cell proliferation period in both *Arabidopsis* and tobacco (*Nicotiana tabacum*) plants (Mizukami and Fischer, 2000). Homologs of *ANT* were investigated in apple fruit and were hypothesized to be involved in cell production during fruit growth (Dash and Malladi, 2012). In this study, two putative homologs of *ANT, MdANT1* and *MdANT2*, were identified. Expression analysis of these genes showed an increased transcript accumulation during early fruit growth when cell division is high. The expression rapidly declined during the translation phase from cell division to cell expansion and remained low throughout the rest of fruit development (Dash and Malladi, 2012). Meanwhile, the high expression of these genes was positively correlated with that of A- and B-type CYC, CDKBs, and DEL1, which are key regulators of cell cycle machinery, suggesting that *ANT* exhibits an integrative coordination with other regulators of cell production that together facilitate fruit growth.

Another floral organ gene that regulates fruit growth and development is the MADX box gene, *SEPALLATA1/2 (SEP1/2)*, which is mainly involved in floral organ identity. In apple, the expression analysis of two *SEP1/2* homologs, *MADS8* and *MADS9*, was performed and their contribution to fruit growth and development was investigated (Ireland et al., 2013). Transgenic apple lines, used in this experiment, showed alteration in floral organs through the production of sepaloid petals, and displayed reduced fruit growth *via* altering the hypanthium development, and exhibited delayed maturity. The cell production within cortex tissue was substantially reduced and cells were considerably smaller, resulting in reduced fruit size. At maturity, fruits from these lines never reach ripening, indicating that *MADS8* and *MADS9* are involved in the regulation of ripening factors such as ethylene synthesis. Such hypothesis was supported by the transient assays indicating that *MADS9* gene is functionally complementary to that of tomato *RIN* gene, and acts as a transcriptional regulator of the ethylene biosynthesis enzyme, 1-aminocyclopropane-1carboxylate (ACC) synthase1 (Ireland et al., 2013). Together, these observations indicate that these genes form a complex network and are involved in multiple aspects of fruit growth and development.

In recent years, the regulation of fruit growth by post-transcriptional regulatory machinery (i.e., microRNA), has intensively been studied. One such microRNA is miRNA172 that positively regulates *Arabidopsis* fruit (siliques) growth and size (José Ripoll et al., 2015). Elevated transcriptional accumulation of miRNA172 resulted in increased fruit size while its reduced expression blocked silique growth. In apple, 75 miRNA families were identified of which 23 were conserved, 10 were less conserved, and 42 were apple-specific (Xia et al., 2012). Of these, miRNA172 was grouped within the conserved family, indicating that it has a general regulatory function in plants. A total of 16 miRNA172 genes (miRNA172a-p) have been identified in apple, from which the post-transcriptional accumulation of only one (miRNA172p) has been confirmed (Yao et al., 2015). In apple, increased transcriptional regulation of miRNA172p, unlike in *Arabidopsis*, negatively influenced fruit growth (Yao et al., 2015). Such contradiction is presumed to be due to the tissue from which the fruit was derived; *Arabidopsis* fruit (siliques) is derived from a developing ovary, whereas apple fruit is derived from the fused basal region of floral appendages (Yao et al., 2015). Overexpression of miRNA172p in a transgenic line of apple reduces fruit of cultivated apple, ‘Royal Gala’, to that of crabapples in size. This negative control was further supported by the transposon insertional allele of miRNA172p, which has a reduced expression of miRNA, and exhibited increased fruit size (Yao et al., 2015). Histological analysis of these transgenic lines indicated that, in addition to reduced fruit size, overexpression of miRNA172p caused alteration in floral organs such as producing flowers with entirely carpel tissue that lack sepals, petals, and stamens. Such modification in floral organs overlapped with that in *Arabidopsis* in which miRNA172p transcriptionally represses the expression of APT2 gene, resulting in floral organ identity defects (Chen, 2004). Additionally, elevated miRNA172p accumulation in the transgenic line of apple resulted in displaying statistically significant thinner hypanthium, thinner cortex tissue, and fewer cells during the cell-division phase, and reduced cell size during the cell-expansion phase (Yao et al., 2015). Together, these developmental data suggest that increased expression of miRNA172p inhibits cell division and cell expansion during early and late developmental stages, respectively, resulting in reduced fruit size.

An important aspect of fruit growth is the hormonal interplay regulating fruit growth in apple. Multiple phytohormones are involved in various aspects of fruit growth and development including cell production, cell expansion, cell-to-cell communication, and fruit ripening (Srivastava and Handa, 2005). Of these, auxin is among the best-studied hormones that plays fundamental roles in fruit growth. In a study investigating the role of auxin in apple fruit size, an auxin signaling-related gene, *ARF106*, was identified to be co-localized with two QTLs associated with fruit size (Devoghalaere et al., 2012). Additionally, cytokinins, abscisic acid (ABA), and ethylene are believed to promote cell production during early fruit growth, fruit abscission, and fruit ripening, respectively (Malladi, 2020). These data indicate that phytohormones play substantial roles in the regulation of fruit growth. Another important but often underappreciated component of fruit growth and development is cell wall hydrolases, which regulate the disassembling of polysaccharidic complexes of the primary cell wall enabling cell enlargement. Cell expansion is primarily mediated by turgor within cells (Cosgrove, 2018), and associated cell wall loosening resulting in an irreversible increase in the surface area (Cosgrove, 2016; Cosgrove, 2018). Cell wall loosening is triggered by enzymes such as expansins, endoglucanases, endotransglucosylases, and pectin‐modifying enzymes such as pectinmethylesterases and polygalacturonases (Cosgrove, 2016). An increased transcriptional regulation of multiple *EXPANSIN (EXPA)* genes during cell expansion indicate that cell wall hydrolases plays an important role in cell enlargement during fruit growth (Dash et al., 2013; Malladi, 2020).

### 5.3 The quantitative inheritance of fruit size

Fruit size is a quantitative trait that is controlled by multiple genetic loci, each with varying effects (Brown, 1960). These multiple and interactive genetic effects make fruit size a difficult trait to study. Such complex traits in quantitative genetic are often studied using QTL mapping. QTL is defined as a given genomic region that contains genes responsible for variation in a quantitative trait in a population (Doerge, 2002; Collard et al., 2005). The fundamental aim of QTL analysis is to identify the genomic regions controlling the phenotypic variation, and to understand how genotype can influence a complex phenotype. Such regions are often located within a broad genomic interval for which subsequent experiments, usually fine mapping or GWAS or a functional genomic approach, are required to generate information about the role of individual genes, and the interactions among them as well as with the environment (Doerge, 2002).

Additional factors that add to the complexity of QTL analysis include the sheer number of QTLs associated with the quantitative trait, the possible epistasis or the interaction between QTLs, and the many additional sources of variation such that environmental stimuli, nutritional composition, field layout, and management practices (Mackay, 2001). One way to overcome this is to reduce the epigenetic sources of variation as much as possible to enhance dissecting the complex phenotype. For instance, the sample of individuals used in the experiment has to be large, usually with an observable amount of recombination. The experimental population is usually derived from homozygous, inbred parental lines in which different alleles at loci associated with variation in the trait of interest are fixed (Mackay, 2001; Doerge, 2002). Hybrid individuals in the F1 population tend to be heterozygous at all markers and QTLs (Doerge, 2002). Further crosses, such as backcrosses, F2 intercross, and crosses to generate recombinant inbred lines (RILs), are made in which molecular markers and QTLs appear to be normally segregated across the chromosomes unless subjected to segregation distortion (Doerge, 2002).

In apple, however, hybridization is restricted to F1 generation because of the self-incompatible nature of the organism (thus, the outcrossed population). Several fundamental distinctions between inbred and outcrossed populations have existed in terms of QTL analysis. For instance, detected QTLs represent between-population variation in inbreds, in which the differences are fixed, whereas outbred populations outline within-population variation (Lynch and Walsh, 1998). Additionally, QTL effects are expressed differently in these populations; typically, as means (the average value of each QTL genotype) in inbreds, and as genetic variances in outbreds. Using within-population variation results in reduced statistical power to detect QTL, and reduced QTL resolution resulting in deteriorate accuracy of estimates from outbred populations mainly because variances are estimated with much less precision than means (Lynch and Walsh, 1998). Such complexity made QTL mapping even more challenging in outbred populations, which requires additional consideration for downstream analysis. Multiple statistical and computational methods have been developed to analyze mapping data and investigate major quantitative trait loci in experimental organisms, including apple (Zeng, 1994). These techniques include single-marker mapping (Edwards et al., 1987; Beckmann and Soller, 1988; Luo and Kearsey, 1989), interval mapping (Lander and Botstein, 1989), composite interval mapping (Jansen, 1993; Jansen and Stam, 1994; Zeng, 1994), and multiple trait mapping (Jiang and Zeng, 1995; Korol et al., 1998; Kao et al., 1999), which facilitates statistical analysis of the associations between phenotype and genotype, and assesses in identifying the genomic regions that are responsible for variation in the quantitative traits.

Recent advances in computational and statistical techniques combined with the molecular markers made it possible to detect QTLs that are responsible for variation in quantitative traits in human, animal, and plant populations (Doerge, 2002). Steven Tanksley and his colleagues conducted a decade-long study using seven wild species of tomato and seven different crosses. They identified 28 QTLs responsible for fruit weight in tomato (Grandillo et al., 1999), some of which show a significant effect on the phenotypic variation. One of these important QTL, *FW2.2*, was further investigated and localized to a narrow genomic region and its contribution to the total variation in fruit size was identified (Alpert et al., 1995; Alpert and Tanksley, 1996). The *FW2.2* QTL is controlled by a single open reading frame, ORFX, which displays an elevated transcript accumulation in the early floral developmental stage in small-fruited wild species (Frary et al., 2000). When the wild-type allele of *FW2.2* was transformed into large-fruited species, fruit size was reduced in size to that of wild-type. This was a revolutionary experiment in QTL mapping, and perhaps the first locus characterized at a molecular level. The increased transcriptional accumulation of *FW2.2* in wild species indicates that it negatively regulates cell production of floral organs leading to reduced fruit size (Frary et al., 2000). The total phenotypic variation controlled by *FW2.2* is approximately 30% among tomato species (Alpert et al., 1995; Frary et al., 2000).

Multiple orthologs of *FW2.2* have been identified in other crops such as maize (Guo et al., 2010), soybean (Libault et al., 2010), avocado (Dahan et al., 2010), sweet and sour cherries (De Franceschi et al., 2013), and pears (Tian et al., 2016). The transcript abundance of these genes is consistent with those of tomato; generally, elevated in small-fruited cultivars during early fruit growth stages (cell division phase), and reduced in large-fruited cultivars, suggesting that *FW2.2* may have been involved in fruit size regulation in a broader magnitude in the eukaryotic gene family. In apple, however, there have been no reports of *FW2.2-like* genes being involved in regulating fruit growth. Hence, we isolated the putative apple orthologs of the *FW2.2* gene from small-, medium-, and large-fruited species at different growth stages and named these genes *Cell Number Regulators* (*CNRs*). These genes showed increased expression during early fruit growth in small-fruited crabapple, associating with reduced relative cell production rate (RCPR). The negatively correlated expression patterns of MdCNRs genes with cell number suggest that alteration in cell number, leading to a subsequent reduction in fruit size is caused by reduced cell division most likely due to changes in CNRs accumulation (unpublished data). These data in conjunction with histological analysis of cell production and cell size, will improve our understanding on the roles of such genes and their contribution to fruit growth.

Additionally, in apple, multiple QTLs responsible for various fruit-quality traits, including fruit size, have been identified (Liebhard et al., 2003; Kenis et al., 2008; Costa, 2015). However, the detected QTLs responsible for qualitative traits typically display reduced resolution and instability against environment, particularly across the multiple years of the experiment. This could perhaps represent the limited access to genomic data at early stage of genome sequencing. As the apple genome was assembled, detailed analysis of such complex traits has been achieved. A total of six genomic regions were identified associated with fruit weight using two F1 hybrid populations, two of which were conserved in both segregated populations (Devoghalaere et al., 2012). The genotypic variation explained by each QTL ranged between 3.9% to 17.3%, depending on the cultivar under consideration. Of these, one QTL was co-localized to a genomic region that contains an auxin response factor (*ARF106*), which displayed high transcript accumulation during the cell division and cell expansion stages, suggesting that the QTL includes genes involved in regulating cell-cycle machineries, leading to increased fruit size (Devoghalaere et al., 2012). In addition to *ARF106*, more than 10 genes related to fruit growth and development, including those involved in cell-cycle regulation, have been identified through QTL mapping (Chang et al., 2014). However, no reports of a major gene underlying fruit size regulation, such as that of tomato *FW2.2* are yet available. In a similar study, two F1 populations were screened for QTLs underlying phenotypic variations in fruit size (Potts et al., 2014). Their findings indicate that variation in fruit size was controlled by two QTLs, that explained 15.4% and 46.4% of observed phenotypic variation, respectively. However, the proposed variation caused by the two detected QTLs appears to be overexpressed mainly because a small number of genotypes were investigated in the experiment (less than 35% of all genotypes produced fruit).

Recently, QTL mapping has been applied to investigate post-transcriptional regulatory mechanisms (i.e., microRNA). Yao and colleagues showed that the crabapple fruit size allele (CAFS), which regulates miRNA172p transcript accumulation was co-localized with a major fruit-size QTL on linkage group11, which explained 13.9% of the phenotypic variation (Yao et al., 2015). As previously explained, miRNA172p negatively regulates apple fruit size, and its expression was reduced when a transposon mutant allele, *cafs*, was introduced, which resulted in increased fruit size in a transgenic apple line. Though the presence of the *cafs* allele in large-fruited apple was suggested to be strongly associated with increased fruit size, it cannot explain all observed variation and therefore other QTLs must contribute to fruit size regulation (Yao et al., 2015). These data represent the movement of QTL studies beyond genomic boundaries and have led to the study of transcriptomic mechanisms, which empower science to obtain a better understanding of the molecular basis responsible for variation in complex traits.

### 5.4 Whole genome approach and fruit growth

Quantitative trait loci mapping and marker-assisted selection (MAS) have been used extensively as the primary methodologies to identify the molecular basis underlying variation in a qualitative trait. Of these, interval mapping has been widely performed using bi-parental families to characterize phenotypic differentiations (Peace et al., 2019). However, despite their extensive implementation, such methods have several limitations. First, genetic variation in the population is limited to those within the two parental strains, thus only a small fraction of the genetic diversity in *Malus* is captured (Peace et al., 2019). Second, MAS is effective solely for identifying large-effected QTLs linked to known markers (Chagné et al., 2007; Bus et al., 2009), and its efficiency is reduced when genes each with small effect are responsible for the variation (i.e., most fruit quality traits including fruit size) (Kumar et al., 2012b). Third, the associations between the marker and trait, and the discovery of QTLs are restricted to the parental cultivars, which exhibits reduced stability across different genetic backgrounds and different environments (Kumar et al., 2012b; Peace et al., 2019). Finally, bi-parental crosses display a reduced recombination rate resulting in poor mapping resolution, such that identifying individual genes with confidence is difficult (Peace et al., 2019).

Some of these limitations can be overcome by identifying single nucleotide polymorphisms (SNPs) across the genome that are underlying the QTLs or by predicting breeding values through whole-genome studies, such as genome-wide association studies (GWAS) and genome selection (GS). Such methods are statistical approaches that maximize identification of SNPs with the assumption that functional alleles will likely exhibit linkage disequilibrium (LD) with at least one of the genotyped markers (Myles et al., 2009; Kumar et al., 2012a). Once SNPs are identified across the genome, their association with the phenotypic variation of the trait is calculated (McClure et al., 2018). In this approach, mostly a collection of diverse individual genotypes or diverse populations that are unrelated and can capture all possible recombination events are used. Such a population structure results in increased mapping resolution, improved transferability from one family to another, and increased discovery of SNPs across the whole genome (Kumar et al., 2012a; Peace et al., 2019; Thapa et al., 2021).

The emphasis on genome-wide studies is on predicting the total genetic value rather than identifying a specific gene and estimating its effect on the phenotypic variation (Kumar et al., 2012a). Thus, the total breeding value (BV), if only additive genetic values are desired, or total genetic value (GV), if additive and non-additive constitutes are desired, are estimated and incorporated into the model (Kumar et al., 2012a). These estimations are performed by producing a single breeding value for each experimental unit *via* obtaining best linear unbiased predictions (BLUP), in which the precision of prediction correlates with the number of observations, heritability of the trait, markers density, and the LD among the markers (Habier et al., 2007; Hayes et al., 2009).

The first draft of the whole-genome sequence of apple, published in 2010, has empowered the research community to explore the whole genome for variant discovery, individual gene identification, and fine mapping (Velasco et al., 2010). In a GS approach searching genomic variation associated with six fruit-quality traits across the whole genome, using a genetically diverse population derived from seven sib-families, a total of 8,000 SNP markers were identified that co-segregate with loci responsible for the phenotypic variation (Kumar et al., 2012b). To validate model accuracy for GV and BV values, two methods were compared, random-regression best linear unbiased prediction (RR-BLUP) and the Bayesian LASSO statistical method (Kumar et al., 2012b). Several conclusions can be drawn from the findings of this experiment. First, because the experimental population was derived from multiple, small sib-families, quantitative loci appear to be more stable across diverse genetic backgrounds. Second, identified genomic regions can be accurately dissected to identify individual genes that contribute to the trait variation. Third, many small-effect loci could be discovered in addition to those large-effect loci previously detected, which increases to the total genetic value responsible for the trait.

A whole-genome approach was also applied to study other economically important traits. Pedersen and colleagues identified 49 fruit volatile organic compounds (VOCs), using gas chromatography-mass spectrometry (GC-MS) analysis of apple juice samples, using 149 diverse apple cultivars (Larsen et al., 2019). Markers associated with these compounds were co-localized into a genomic region rich in several alcohol acyltransferases, including AAT1. Similarly, an association analysis, using 162 possibly unrelated apple accessions, identified two QTLs associated with volatiles interplaying with fruit texture (Farneti et al., 2017). Additionally, SNPs markers for sugar compositions (e.g., sucrose and fructose) were identified on chromosome 1, which explained 24% and 47% of the variation, respectively. Finally, markers controlling harvest date were co-localized with the coding region of a NAC transcription factor that regulates fruit ripening and maturity in apple (Larsen et al., 2019). Recently, a genome-wide methods was applied to study other physiological traits such as fruit texture (Di Guardo et al., 2017), fire blight resistance loci (Thapa et al., 2021), scab resistance (McClure et al., 2018), and flesh browning (Kunihisa et al., 2021). Findings of these studies indicate that genome-wide techniques can facilitate increased mapping resolution, increased detection of multiple alleles at the same locus, and a high number of SNPs that adequately cover the whole genome. However, no reports on investigating fruit size using genome-wide studies, are yet available. Thus, we performed an association study to increase the precision and improve the stability of QTL analyses. We performed multiple quantitative genetic analyses to elucidate the underlying genetic architecture of fruit mass. Our approach encompasses different strategies for association studies to identify regions containing QTLs, comparing different cross-validation scenarios, performing genomic covariance analysis to investigate trait stability across years, as well as to examine pleiotropy between fruit mass and other physiological traits influencing fruit mass. A total of nine genomic regions associated with fruit mass were identified, two of which are novel to this study, markers Md14_26050918 and Md14_26050904. Detected QTLs explained ~ 42% of the total genetic variation of which ~ 20% is explained by the two novel QTLs. Regions responsible for fruit mass variation appeared to be under strong additive and epistatic genetic control. These regions exhibited high stability across families, as well as across years, and showed accurate genomic prediction across families (Jahed and Hirst, unpublished data).

### 5.5 Epigenetic regulation of fruit growth

In population genetics, phenotype is defined as the result of genotype and its interactions with the environment. In previous sections, we discussed the molecular, physiological, and genomics components of fruit growth and development. An equally important aspect of the process is the epigenetic dimension. Since apple is clonally propagated, over time this could lead to epimutation, typically through DNA methylation events, which may influence different phenotypes (Daccord et al., 2017). Therefore, understanding the epigenetic background could provide valuable information for studying somatic variation, leading to developing epigenetic markers for downstream analysis (Peace et al., 2019). At the molecular level, epigenetics is defined as any change in transcript accumulations that is caused by factors other than DNA sequence manipulation. To investigate epigenetic events, a trait under consideration must display minimum genetic changes in the genome. This is particularly challenging for polygenic traits such as fruit size, as it is difficult to simultaneously control all genes responsible for trait variation. A proposed organ for such epigenetics studies is sports (i.e., clones exhibiting novel phenotypes), which usually display few genetic changes (Peace et al., 2019). However, some sports might be produced as a result of rare genetic changes, such as those induced by transposable elements (Han et al., 2017), or might be regulated by DNA methylation (Peace et al., 2019). The methylome dynamics of early fruit was the first attempt to understand the epigenetic mechanisms underlying fruit size (Daccord et al., 2017). These findings suggest that apple fruit development is a complex trait in which epigenetics play a critical role. These authors also provided a comprehensive list of putative genes involved in epigenetic regulation of fruit growth and development (Daccord et al., 2017). These data indicate that investigating epigenetic events and their contribution to trait variation is important; however, studying complex traits will likely be difficult.

### 5.6 The role of domestication in apple fruit size

Plant domestication has largely contributed to the process of fruit growth and development leading to increased fruit size during which beneficial alleles underlying yield and quality have been selected by mammals including humans who have acted as distribution vehicles (Harada et al., 2005; Paran and van der Knaap, 2007). Selection for traits of interest during domestication can be classified and characterized in several ways (Wedger et al., 2021). One way of classification is based on phenotypic diversification and is divided into two types: directional selection where variation in selected trait decreases, and diversifying selection in which phenotypic variation increases (Meyer and Purugganan, 2013). Under this classification, fruit size is subject to directional selection in which underlying genes tend to show reduced diversity in selected population, compared with its wild progenitor, mainly because alleles that are responsible for the trait variation are fixed (Ross-Ibarra et al., 2007).

An alternative mode of classification is based on human involvement in the domestication process and their coevolutionary relationship with plants (Zeder, 2006). This classification system encompasses two phases: an initial phase in which human were unconsciously involved in selection during their early interactions with plants (Zohary, 2004), followed by a subsequent, intentional manner to consciously select traits for improved breeding values (Duan et al., 2017; Wedger et al., 2021). In this context, fruit size is thought to be under unconscious selection, where large-fruited cultivars were unconsciously selected early in the domestication process, leading to reduced phenotypic variation among the selected species (Kluyver et al., 2013; Cunniff et al., 2014; Kluyver et al., 2017; Purugganan, 2019). Such selecting discrimination tends to result in selective sweeps at loci controlling fruit size, leading to genetic bottlenecks, and result in reduced genetic diversity in the population (Duan et al., 2017; Wedger et al., 2021). However, recent whole genome analysis showed that cultivated apple has been hybridized with the Caucasian and European crabapple, *M. sylvestris*, during apple dispersal from Asia to Europe along the Silk Road (Cornille et al., 2014; Peace et al., 2019), from which beneficial alleles have been introduced into cultivated apples. Such crop-to-wild hybridization resulted in successful introgression of the high diversity that is present in domesticated apple (Peace et al., 2019).

Recently, genome-wide study and candidate-gene approaches revealed insights into the evolution of fruit developmental aspects during the domestication process and the introgression due to hybridization with *M. sylvestris*. A two-stage model for fruit enlargement during domestication, using a whole-genome approach was proposed (Duan et al., 2017). This model suggests that while domesticated apple originated from a relatively large-fruited species, *M. sieversii*, whose genome has been reshaped by crossing to *M. sylvestris*, a major secondary contributor of the cultivated apple. Introducing genomic information from the secondary progenitor into domesticated apple suggests that apple has been under much lower evolutionary pressure compared to other crops and is one of the primary factors involved in maintaining diversity in cultivated apple (Duan et al., 2017). In a similar approach, two subset of candidate domestication genes, one displaying very high diversity, and another exhibiting low diversity, were identified in cultivated apple (Wedger et al., 2021). Of these, the low-diversity genes were incorporated into traits that are predicted to be under conscious selection, such as color and flavor, while the high-diversity genes were involved in several other quality traits. Their findings were consistent with those showing that apples benefited greatly from the intensive introgression from *M. sylvestris*. Taken together, these data indicate that apple fruit growth is under a tight control of both endogenous and exogenous factors, which make it a complex multifactorial inherited trait.

## 6 Limitations associated with quantitative genetic approach in apple and specific recommendations

### 6.1 The experimental population

The statistical power to detect genomic regions associated with fruit size is determined by several factors including the number of individuals in the mapping population, the recombination frequency, and the marker density on a chromosome (Mauricio, 2001). Increasing marker density can be achieved by increasing the total number of markers, which is typically associated with an increased number of individuals in the mapping population as well as genetically diverse individuals at a given locus. As the population size increases, the estimated genetic distance between markers is reduced (Tourrette et al., 2021), which best represents the increased recombination rate (Doerge, 2002). The recommended population size to detect a QTL with increased precision and high statistical power is over 500 individuals (Mauricio, 2001). Although such techniques have been used in model plants, their applications to outcrossing species, including apple, is not currently possible, simply because their biological properties prevent the generation of RILs and any advanced crosses (Wu et al., 2010). Additionally, generating a large experimental population in apple is time consuming and expensive. To reduce the time required to generate apple seedlings, one method is to create double haploid (DH) trees, which recently has been performed in apple (Daccord et al., 2017). A DH individual has two genetically identical homologous chromosomes, in which the recombination information is equivalent to that in a backcross population.

### 6.2 Identifying genes underlying a QTL: a needle in haystack

As previously discussed, a QTL represents a genomic region, which encompasses many small-affected loci (i.e., 28 QTLs controlling fruit size in tomato), most of which have small effect on the variation of the quantitative trait. In most cases, these QTLs are intercorrelated, and display a tight interaction with environmental stimuli (Mauricio, 2001). Additionally, variation in fruit size is more likely to be controlled by several genes, each displaying a small overall contribution to the phenotypic differences. Thus, understanding the physiological function of all potential genes in the QTL seems difficult, and will take several years to investigate (Mauricio, 2001). An example is the *FW2.2* gene in tomato, which is responsible for approximately 30% of fruit size variation, which took more than 10 years to characterize at the molecular level (Alpert et al., 1995; Frary et al., 2000). Dissecting the remaining QTLs and determining all the genes influencing fruit size in tomato is a daunting task (Mauricio, 2001). Because of the outcross feature of apple, dissecting QTLs and identifying individual genes imposes additional challenges, particularly if the parental strains do not display alternate alleles. A possible method may be the identification of the genes underlying the QTL region, to date offered by the availability of the apple genome.

### 6.3 Sequencing choice

Despite the recent advances and price reduction in sequencing technology, sequencing a larger number of populations remains difficult. Trading off quality with quantity of sequencing can be challenging. Statistically, larger population sizes lead to increased analysis accuracy. However, sequencing a larger number of individuals can be expensive, thus three approaches are often used: (i) pooling multiple samples and sequence at high sequencing depth, (ii) sub-divide the population into small groups and select representatives from each sub-group at high sequencing depth, or (iii) sequence a larger number of individuals at low sequencing depth. In an association study we conducted, we used the third option; where all individuals were sequenced at low sequencing depth (~ 2X – 10X depth), using the genotyping-by-sequencing (GBS) method. This sequencing method resulted in producing reads with depth as low as 2X, which is challenging for genotyping software to accurately identify a SNP, resulting in the filtering out of a large amount of data. An alternative approach may be an interinstitutional collaboration to sequence a relatively large population at sequencing depth of > 10X to improve SNPs calling accuracy. These genotypic data can be associated with various phenotypic traits.

## 7 Conclusion and future perspectives

**Figure 3:**
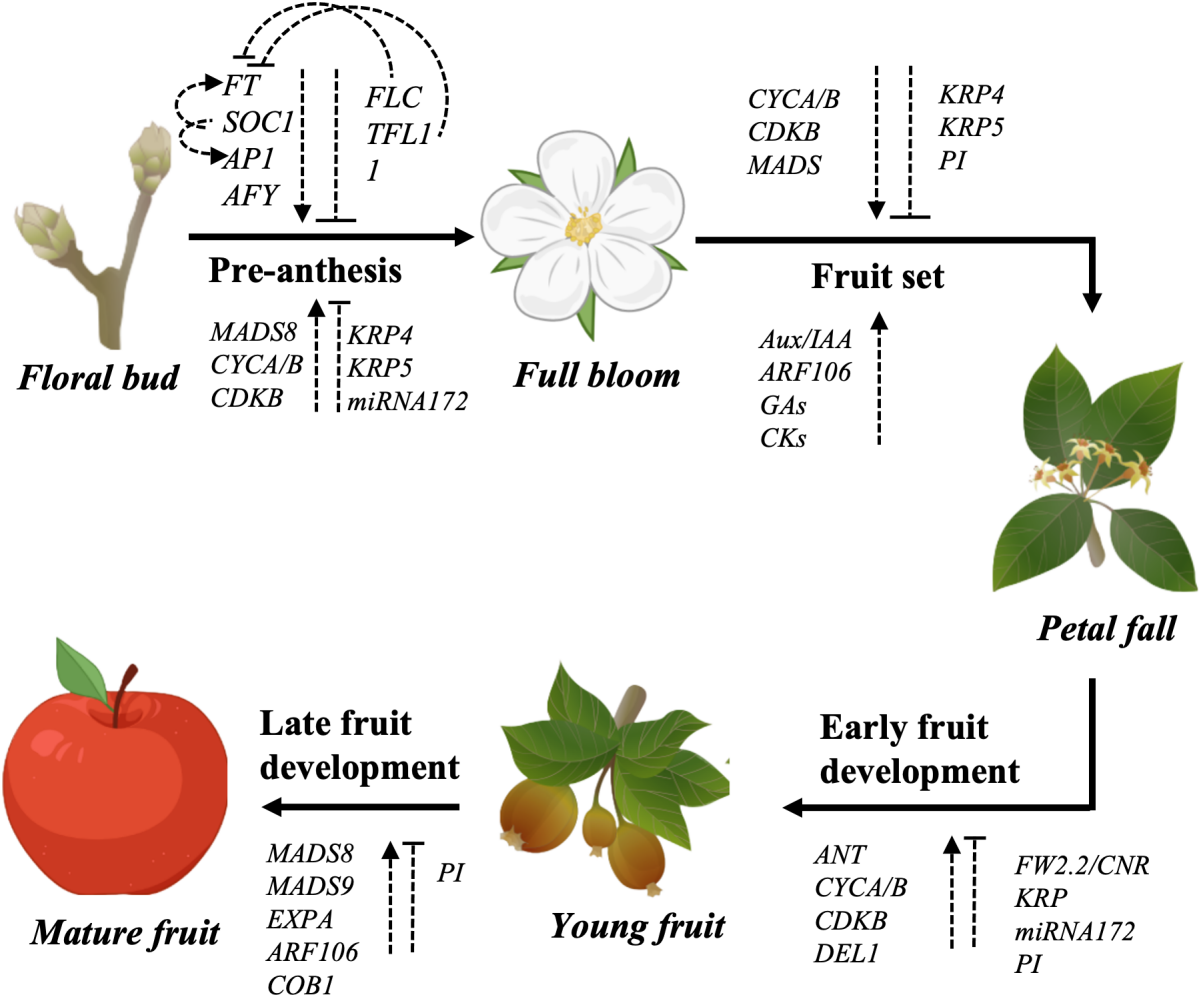
A comprehensive summary of the currently known regulatory factors controlling different fruit developmental stages. Arrows indicate positive regulation while dashed line with a perpendicular line at the end indicates negative regulation.

Fruit size is a complex and multiallelic trait controlled by multiple internal and external factors. Final fruit size is the result of many developmental events occurring in a precise chronological order over the course of a growing season. Understanding the underlying mechanisms by which each event is mediated, requires considerable thought and experimentation. In general, apple fruit development is divided into two distinct stages: the earlier developmental stage ranges from floral bud initiation to fruit set, and the later developmental stage that includes fruit growth, fruit maturity and ripening. Each stage is tightly controlled by a complex network of internal and external factors. A summary of the regulatory factors of each stage is presented in **Figure 3**. The quantitative and complex inheritance and the involvement of different genes make fruit size a quantitative trait. These characteristics add to the challenge of identifying all genes controlling the trait. However, a QTL or GWAS or GS approach can be employed to identify the underlying loci that co-segregate with fruit size.

Over the past decade, considerable advances have been made in producing copious amounts of genomic data for multiple apple cultivars and species. The whole-genome sequence facilitated identifying genomic information responsible for variation in traits of interest including fruit quality (Peace et al., 2019). Several economically important traits have been dissected into individual genes that influence phenotypic differentiation. However, understanding the physiological function of all candidate genes is challenging (Mauricio, 2001). Additionally, most of the QTL mapping utilized bi-parental populations in which the genetic variation is limited to those within the two parental strains, leading to reduced stability when applied to different genetic backgrounds (Kumar et al., 2012b; Peace et al., 2019). An alternative approach can be using a collection of possibly unrelated individuals using an association study approach. We performed an association study using hybrids between distantly related apple species to increase the precision and improve the stability of QTL analyses. We performed multiple quantitative genetic analyses to elucidate the underlying genetic architecture of fruit mass. Our approach encompasses different strategies for association studies to identify regions containing QTLs, comparing different cross-validation scenarios, performing genomic covariance analysis to investigate trait stability across years, as well as to examine pleiotropy between fruit mass and other physiological traits influencing fruit mass.

Additionally, most of the quantitative trait loci work in apple has ended at a predicted candidate genes list. A substantial portion of such information comes either from model organisms or from guesswork. To characterize these genomic regions at the molecular level, further investigation such as using a functional genomic approach is required. However, such approaches require a broader interdisciplinary collaboration of plant biologists, plant molecular biologists, geneticists, and bioinformaticians. Forming multi-institutional and perhaps international collaborations will facilitate the exchange of knowledge and progress towards these goals. Another field of future research could be other “omics” approaches, such as transcriptomics, proteomics, and metabolomics, which have tremendous potential for further dissecting traits of interest and obtaining meaningful knowledge regarding the regulation of apple improvement. The availability of massive genomic data from several apple species, including wild relatives, empowers the research community to acquire valuable information from “omics” mechanisms.

## Author contributions

KRJ conducted research and wrote the initial manuscript draft. PMH provided oversight and edits to improve the manuscript. All authors contributed to the article and approved the submitted version.
